# Moxetumomab pasudotox in relapsed/refractory hairy cell leukemia

**DOI:** 10.1038/s41375-018-0210-1

**Published:** 2018-07-20

**Authors:** Robert J. Kreitman, Claire Dearden, Pier Luigi Zinzani, Julio Delgado, Lionel Karlin, Tadeusz Robak, Douglas E. Gladstone, Philipp le Coutre, Sascha Dietrich, Mirjana Gotic, Loree Larratt, Fritz Offner, Gary Schiller, Ronan Swords, Larry Bacon, Monica Bocchia, Krimo Bouabdallah, Dimitri A. Breems, Agostino Cortelezzi, Shira Dinner, Michael Doubek, Bjorn Tore Gjertsen, Marco Gobbi, Andrzej Hellmann, Stephane Lepretre, Frederic Maloisel, Farhad Ravandi, Philippe Rousselot, Mathias Rummel, Tanya Siddiqi, Tamar Tadmor, Xavier Troussard, Cecilia Arana Yi, Giuseppe Saglio, Gail J. Roboz, Kemal Balic, Nathan Standifer, Peng He, Shannon Marshall, Wyndham Wilson, Ira Pastan, Nai-Shun Yao, Francis Giles

**Affiliations:** 1 National Cancer Institute, National Institutes of Health, Bethesda, MD USA; 2 The Royal Marsden NHS Foundation Trust, London, UK; 3 Institute of Hematology, Seràgnoli University of Bologna, Bologna, Italy; 4 Hospital Clinic, Barcelona, Spain; 5 Centre Hospitalier Lyon Sud, Pierre-bénite, France; 6 Medical University of Lodz, Copernicus Memorial Hospital, Lodz, Poland; 7 Johns Hopkins Kimmel Cancer Center, Baltimore, MD USA; 8 Charité Universitätsmedizin, Berlin, Germany; 9 Universitätsklinikum Heidelberg, Heidelberg, Baden-Württemberg Germany; 10 Clinical Center of Serbia, Belgrade, Serbia; 11 University of Alberta, Edmonton, Alberta Canada; 12 Ghent University Hospital, Ghent, Belgium; 13 David Geffen School of Medicine, UCLA, Los Angeles, CA USA; 14 Sylvester Comprehensive Cancer Center, University of Miami, Miami, FL USA; 15 St. James’s Hospital, Dublin, Ireland; 16 Azienda Ospedaliera Universitaria, University of Siena, Siena, Italy; 17 Service d’hématologie, CHU Bordeaux, F-33000 Bordeaux, France; 18 Ziekenhuis Netwerk Antwerpe, Antwerp, Belgium; 19 Fondazione IRCCS Ca’ Granda Ospedale Maggiore Policlinico, University of Milan, Milan, Italy; 20 Northwestern Medicine Feinberg School of Medicine, Chicago, IL USA; 21 Masaryk University, Brno, Czech Republic; 22 Helse Bergen HF Haukeland University Hospital, Bergen, Norway; 23 Clinic of Hematology, Ospedale Policlinico San Martino, Genova, Italy; 24 Department of Hematology and Transplantation, Medical University of Gdańsk, Gdańsk, Poland; 25 Inserm U1245 and Department of Hematology, Centre Henri Becquerel and Normandie Univ UNIROUEN, Rouen, France; 26 SOL, Clinique Sainte-Anne, Strasbourg, France; 27 MD Anderson Cancer Center, Houston, TX USA; 28 Centre Hospitalier de Versailles, INSERM U1173 Le Chesnay, France; 29 Université Versailles Saint-Quentin-en-Yvelines, Paris Saclay, France; 30 Justus-Liebig University, Giessen, Germany; 31 City of Hope National Medical Center, Duarte, CA USA; 32 Bnai Zion Medical Center, Haifa, Israel; 33 L’hôpital Côte de Nacre, Caen Cedex 9, Caen, France; 34 University of New Mexico, Albuquerque, NM USA; 35 University of Turin, Turin, Italy; 36 Weill Cornell Medicine, The New York Presbyterian Hospital, New York, NY USA; 37 MedImmune, South San Francisco, CA USA; 38 MedImmune, Gaithersburg, MD USA; 39 Developmental Therapeutics Consortium, Chicago, IL USA

## Abstract

This is a pivotal, multicenter, open-label study of moxetumomab pasudotox, a recombinant CD22-targeting immunotoxin, in hairy cell leukemia (HCL), a rare B cell malignancy with high CD22 expression. The study enrolled patients with relapsed/refractory HCL who had ≥2 prior systemic therapies, including ≥1 purine nucleoside analog. Patients received moxetumomab pasudotox 40 µg/kg intravenously on days 1, 3, and 5 every 28 days for ≤6 cycles. Blinded independent central review determined disease response and minimal residual disease (MRD) status. Among 80 patients (79% males; median age, 60.0 years), durable complete response (CR) rate was 30%, CR rate was 41%, and objective response rate (CR and partial response) was 75%; 64 patients (80%) achieved hematologic remission. Among complete responders, 27 (85%) achieved MRD negativity by immunohistochemistry. The most frequent adverse events (AEs) were peripheral edema (39%), nausea (35%), fatigue (34%), and headache (33%). Treatment-related serious AEs of hemolytic uremic syndrome (7.5%) and capillary leak syndrome (5%) were reversible and generally manageable with supportive care and treatment discontinuation (6 patients; 7.5%). Moxetumomab pasudotox treatment achieved a high rate of independently assessed durable response and MRD eradication in heavily pretreated patients with HCL, with acceptable tolerability.

## Introduction

Patients with hairy cell leukemia (HCL), a rare B cell malignancy characterized by high CD22 expression, typically present with pancytopenia and increased susceptibility to infection [1]. Although many patients achieve long-term complete remission with the purine nucleoside analogs pentostatin or cladribine [2–4], ~50% will relapse by 16 years and require additional treatment [5]. In later lines, purine nucleoside analogs offer lower complete response rate, shorter duration of response, and higher risk of cumulative toxicity compared with earlier treatment [5–7]. Of note, purine nucleoside analogs are associated with severe infection due to profound neutropenia [8]. Targeted therapies, such as vemurafenib, ibrutinib, and rituximab, show encouraging efficacy but rarely eradicate minimal residual disease in patients with complete response as single agents, and are often associated with safety and tolerability concerns [5, 9–18]. An unmet need remains in relapsed/refractory HCL for therapies that provide durable complete response with eradication of minimal residual disease and less myelo/immunosuppression.

Moxetumomab pasudotox (CAT-8015) is a recombinant immunotoxin targeting CD22, composed of an immunoglobulin light chain variable domain and a heavy chain variable domain genetically fused to a truncated form of *Pseudomonas* exotoxin PE38 [19]. In a phase 1 study of patients with relapsed/refractory HCL, 49 patients received moxetumomab pasudotox [20]. The objective response rate was 86%, with a complete response rate of 57%; 63% of patients who achieved complete response were minimal residual disease negative by immunohistochemistry as assessed by an independent central reviewer, and similar percentage by flow cytometry, either of which translated into longer duration of complete response and/or progression-free survival [20, 21]. The current study evaluated the rate of durable complete response with moxetumomab pasudotox in patients with multiply relapsed HCL.

## Methods

### Study design and organization

This pivotal, multicenter, single-arm open-label study (clinicaltrials.gov identifier: NCT01829711) was conducted at 32 centers in 14 countries. The study was performed in accordance with the principles of the Declaration of Helsinki, the International Conference on Harmonisation/Good Clinical Practice guidelines, and applicable regulatory requirements. The protocol was approved by the institutional review board at each center. All patients provided written informed consent.

The authors designed the study in collaboration with the sponsor. Data were collected and analyzed by the sponsor and interpreted jointly with the authors. All authors had full access to the data. The first draft was written by the authors and Peloton Advantage, who also provided editorial support. All authors reviewed and contributed to subsequent drafts and vouch for the completeness and veracity of the data and analyses, and for adherence to the protocol.

### Patients

Adults with histologically confirmed HCL and an indication for treatment (defined as at least one of the following: neutrophils <1.0 × 10^9^/L, platelets <100 × 10^9^/L, hemoglobin <10 g/dL, or symptomatic splenomegaly) were eligible. Patients must have received at least two prior systemic therapies, including two courses of a purine nucleoside analog or one course of rituximab or a *BRAF* inhibitor following a single prior purine nucleoside analog course. An Eastern Cooperative Oncology Group performance status of 0, 1, or 2 and adequate hepatic and renal function were required. Detailed inclusion and exclusion criteria are shown in the protocol.

### Study treatment

Patients received moxetumomab pasudotox 40 μg/kg intravenously over 30 minutes on days 1, 3, and 5 of 28-day cycles for a maximum of six cycles or until documentation of minimal residual disease-negative complete response (as assessed by the investigator), disease progression, initiation of alternate therapy, or unacceptable toxicity. Patients received prophylaxis for renal insufficiency (fluids and low-dose aspirin) and hypersensitivity reactions (hydroxyzine, acetaminophen, and ranitidine; see protocol for details).

### Study end points and assessments

The primary end point was durable complete response, defined as complete response assessed by blinded independent central review with maintenance of hematologic remission for more than 180 days. Complete response was defined based on pathology (no evidence of hairy cells in bone marrow by routine hemoxylin and eosin stain), imaging (resolution of splenomegaly, hepatomegaly, and lymphadenopathy, documented by CT or MRI), and normalization of hematologic parameters (neutrophils ≥1.5 × 10^9^/L, platelets ≥ 100 × 10^9^/L, and hemoglobin ≥ 11.0 g/dL without growth factors or transfusions in 4 weeks). Relapse was defined as loss of any criteria needed for best response, including asymptomatic reappearance of hairy cells in the bone marrow by hemoxylin and eosin stain. Additional details are described in the protocol. Secondary efficacy end points included objective response rate, duration of complete and objective response, progression-free survival, safety/tolerability, immunogenicity, and pharmacokinetics.

Minimal residual disease was independently assessed using immunohistochemistry. A blinded independent pathologist assessed bone marrow biopsy specimens stained for the HCL/B cell antigens CD20, CD79a, Annexin A1, DBA.44, and PAX-5; additional details are in the Supplementary Appendix. During treatment, minimal residual disease was assessed locally at study sites by flow cytometric analysis of peripheral blood and/or bone marrow aspirate, according to each site’s procedures.

Safety assessments included adverse events, serious adverse events, and changes in clinical laboratory evaluations and vital signs through 30 days after the last moxetumomab pasudotox dose. Adverse events and serious adverse events were assessed by the investigators for relationship to moxetumomab pasudotox, graded using National Cancer Institute Common Toxicity Criteria for Adverse Events V4.03 and coded using Medical Dictionary for Regulatory Activities V20.0.

Plasma moxetumomab pasudotox concentrations were assessed at multiple time points following dosing. Pharmacokinetic parameters were estimated by non-compartmental approach using Phoenix^®^ WinNonlin^®^ (Version 6.3, Certara, Princeton, New Jersey). Immunogenicity was evaluated at multiple time points. Samples that were positive for anti-drug antibodies were evaluated for neutralization, specificity (PE38 versus CD22 binding domain), and titer. Pharmacodynamics were assessed by measuring peripheral blood B cell counts (CD19 + B cells which include hairy cells) at multiple time points. Further details are provided in the protocol.

Efficacy was evaluated in the intent-to-treat population, which included all patients who entered and received moxetumomab pasudotox, and safety was evaluated in the safety population, which comprised all patients who received at least one dose of moxetumomab pasudotox; both populations comprise the same 80 patients.

### Statistical analysis

Rituximab was used as a historical control to determine sample size, as it was the most frequently used nonchemotherapy treatment in relapsed/refractory HCL with a CR rate of 13% in the largest study [12, 22]. A sample size of 77 patients was planned to provide 90% power to detect a difference between 13% and 28% in durable complete response rates using a 2-sided significance level of 0.05. Durable complete response rate was constructed using the exact probability method (Clopper–Pearson exact interval). If the lower bound of the 95% CI was above 13% (or equivalently, the binomial exact test one-sided *p*-value < 0.025), it is concluded that the durable complete response rate was significantly higher than the historical control value of 13%.

Duration of complete response, duration of objective response, and progression-free survival were estimated with the use of the Kaplan–Meier method. In the analysis of duration of complete response and objective response, patients alive with no documented relapse prior to data cut-off, dropout, or initiation of alternative anticancer therapy were censored on the date of last disease assessment or hematologic assessment, whichever occurred last. In the analysis of progression-free survival, patients alive with no documented relapse or disease progression prior to data cut-off, dropout, or the initiation of alternative anticancer therapy were censored on the date of last disease assessment or hematologic assessment, whichever occurred last.

### Role of funding source

This study and manuscript were funded by MedImmune, the global biologics R&D arm of AstraZeneca. MedImmune employees were involved in the study design, the collection, analysis, and interpretation of data, the review of the manuscript, and the decision to submit for publication.

## Results

### Patients

The database was locked on 24 May 2017. Eighty patients were enrolled and treated (Table 1); 50 (62.5%) patients completed six cycles of treatment and 12 (15.0%) discontinued early after achieving complete response with negative minimal residual disease (Figure S1 in Supplementary Appendix). Enrolled patients had received a median of three lines of prior therapy; 70 patients (87.5%) received at least two lines of purine nucleotide analogs, 60 patients (75.0%) received prior rituximab and 14 patients (17.5%) received prior BRAF-inhibitor (Table S1 in Supplementary Appendix).

**Table 1:** Demographics and baseline characteristics

Characteristic	Value (*N* = 80)
Age	
Median, *y*	60.0
Range, *y*	34–84
Race (excluding patients enrolled in France [*n* = 8]	
White, *n* (%)	70 (97.2)
Black, *n* (%)	1 (1.4)
Asian, *n* (%)	1 (1.4)
Ethnicity (excluding patients enrolled in France [*n* = 8]	
Hispanic or Latino, *n* (%)	4 (5.6)
Not Hispanic or Latino, *n* no. (%)	67 (93.1)
Unknown, *n* no. (%)	1 (1.4)
Variant hairy cell leukemia—no. (%)	3 (3.8)
Splenectomy—no. (%)	5 (6.3)
Eastern Cooperative Oncology Group performance status—no. (%)	
0	49 (61.3)
1	29 (36.3)
2	2 (2.5)
Extent of HCL	
Median hemoglobin (range), *g/dL*	11.10 (6.5, 16.3)
Median neutrophil count (range), *nL*	0.81 (0.1, 6.2)
Median platelet count (range), *nL*	68 (6, 350)
Median hairy cell involvement in bone marrow, % (range)^a^	85 (0, 100)
Median size of spleen, excluding splenectomy, *cm* (range)	13.3 (8.9, 24.7)
Prior cancer therapy	
Median number of lines of prior therapy (range)	3.0 (2, 11)
>3 prior lines, *n* (%)	39 (48.8)
Prior purine nucleoside analog, *n* (%)	80 (100)
Prior rituximab, *n* (%)	60 (75.0)

^a^Determined by blinded independent pathologist read of hematoxylin and eosin stained slides. Two patients were reported as not having baseline bone marrow involvement. In one case there were technical problems with the hematoxylin and eosin stained slides but 90% involvement by immunohistochemistry; in the other case the patient had variant HCL presenting as splenomegaly (182 mm)

### Efficacy

**Fig. 1:**
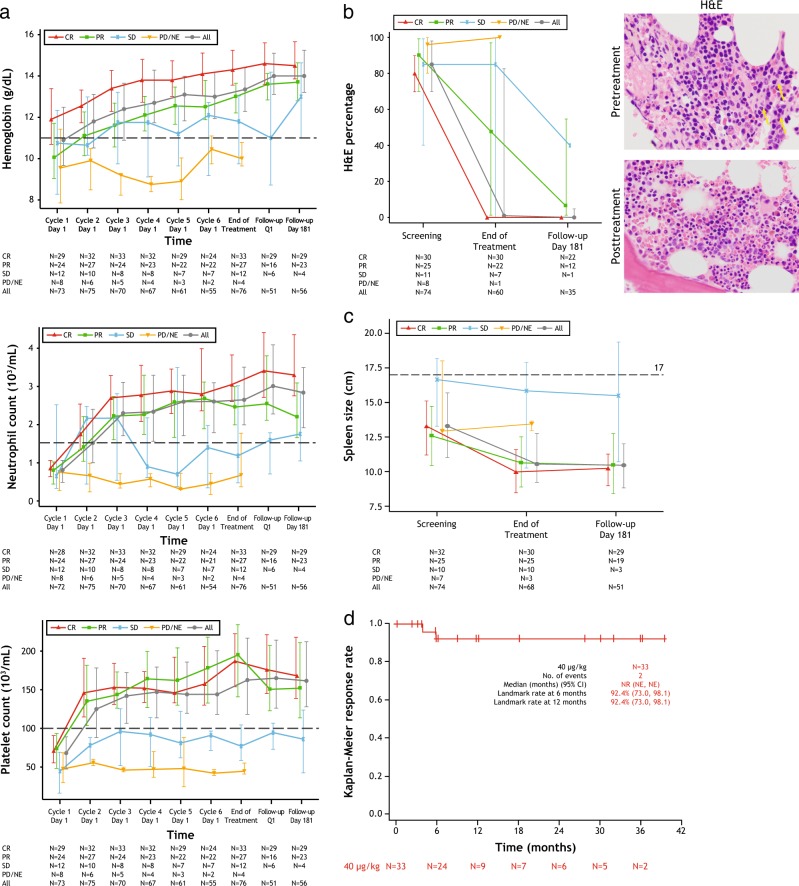
Evaluation of primary end point (blinded independent central review). **a** Hemoglobin (top), neutrophil counts (middle), and platelet counts (bottom) over time, as a function of best objective response. Median and interquartile range are shown at each displayed time point and the threshold for hematologic remission is indicated as a dotted line. **b** HCL involvement in bone marrow, assessed by hematoxylin and eosin stain, as a function of best objective response; median and interquartile range are shown (top), and as representative pretreatment and posttreatment images (×100) of a patient who obtained a minimal residual disease-negative complete response (bottom). Yellow arrows indicate hairy cells. **c** Spleen size by imaging, as a function of best objective response. Median and interquartile range are shown. Splenectomy patients (*n* = 5) not included. **d** Kaplan–Meier plot of duration of hematologic remission from complete response

Median hemoglobin, neutrophil count, and platelet count improved rapidly during treatment (Fig. 1a); 80% of patients (64/80) achieved hematologic remission in about one month (median 1.1 months, 95% CI, 1.0 to 1.2). At a median follow-up of 16.7 months [2.1 to 48.8]), the durable complete response rate was 30% (24/80 patients; 95% CI, 20.3 to 41.3), and the objective response rate was 75% (60/80 patients, 95% CI, 64.1 to 84.0; Table 2) based on blinded independent central review. The complete response rate was 41% (33/80 patients; 95% CI, 30.4 to 52.8). Thirty-three patients achieved complete response, including elimination of leukemic cells in bone marrow by morphologic assessment; significant (>90%) reductions in bone marrow involvement were also observed in 29.6% of patients achieving partial response (8/27; Fig. 1b). Average spleen size decreased during treatment, and among patients with baseline splenomegaly (17 cm or larger), 6 (42.9%) resolved to 14 cm or smaller (Fig. 1c).

**Table 2:** Disease response and minimal residual disease status by immunohistochemistry

Parameter	Value (*N* = 80)	
	**Blinded independent central review**	**Investigator assessment**
Durable complete response (primary end point), *n* (%)^a^	24 (30.0)	38 (47.5)
95% confidence interval	20.3, 41.3	36.2, 59.0
Best overall response^a^		
Complete response, *n* (%)	33 (41.3)	41 (51.3)
95% confidence interval	30.4, 52.8	39.8, 62.6
Complete response, minimal residual disease negative, *n* (%)	27 (33.8)	26 (32.5)
95% confidence interval	23.6, 45.2	22.4, 43.9
Partial response, *n* (%)	27 (33.8)	27 (33.8)
Objective response rate (complete or partial response), *n* (%)^a^	60 (75.0)	63 (78.8)
95% confidence interval	64.1, 84.0	68.2, 87.1

^a^Two-sided confidence interval was calculated using the exact probability method based on the binomial distribution

**Fig. 2:**
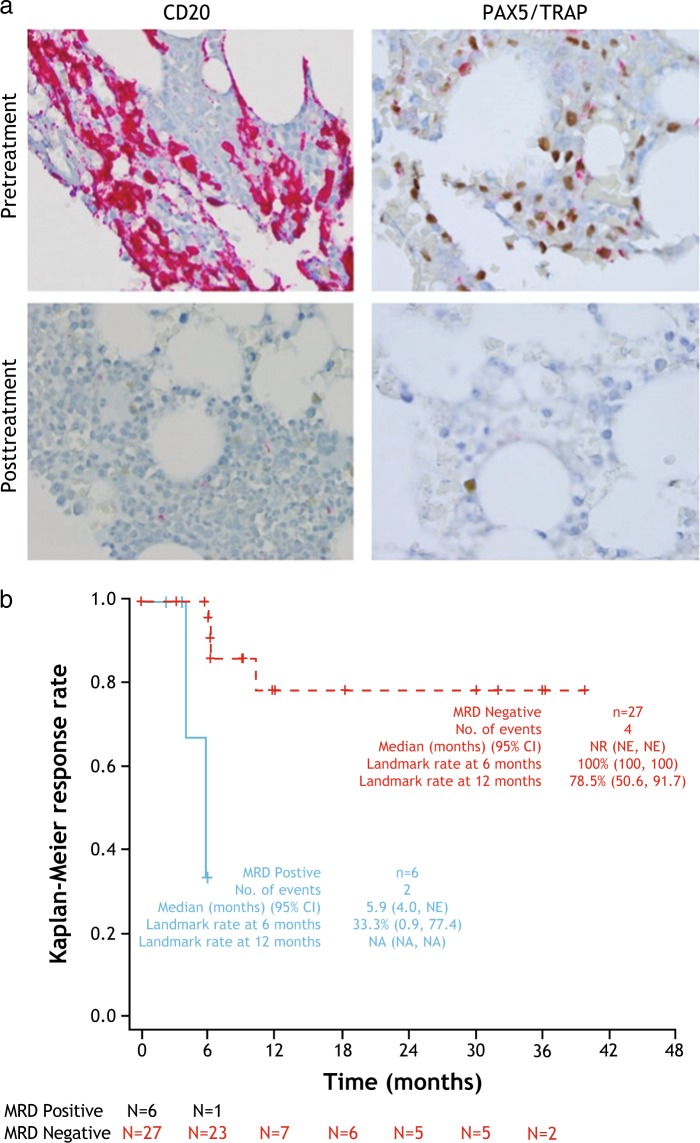
Assessment of minimal residual disease by immunohistochemistry (blinded independent central review). **a** Representative immunohistochemistry images from pretreatment and posttreatment bone marrow biopsy specimens of the same patient shown in Fig. 1b: CD20 (left) and PAX5/TRAP (right). **b** Kaplan–Meier plot of duration of complete response, by minimal residual disease status

Most (28/33) complete responses were achieved at the end of treatment disease assessment; five patients achieved complete response at disease assessment six months after the end of treatment. The median duration of hematologic remission from complete response (Fig. 1d), median duration of complete response, and median progression-free survival were not reached. Six patients relapsed from complete response as of the data cut-off; four had asymptomatic relapse with only reappearance of hairy cells in the bone marrow with normal hematological counts and two had loss of hematologic remission. Among complete responders, 27 (85%) patients achieved minimal residual disease negativity as assessed by immunohistochemistry (Table 2 and Fig. 2). The median duration of complete response for minimal residual disease-positive patients was 5.9 months and was not reached for minimal residual disease-negative patients (Fig. 2b). Subgroup analyses are presented in Figure S2 in the Supplementary Appendix.

### Safety

The most common adverse events were peripheral edema (38.8%), nausea (35.0%), and fatigue (33.8%) (Table 3); the most common treatment-related adverse events were nausea (27.5%), peripheral edema (26.3%), headache (21.3%), and pyrexia (20.0%). The most common treatment-related grade 3/4 adverse events were decreased lymphocyte count (7.5%) and hemolytic uremia syndrome (5.0%). Grade 3 or 4 infections occurred in 13 (16.3%) patients, with infections in 2 patients (2.5%) reported as treatment related. Serious adverse events in at least 5% of patients were hemolytic uremic syndrome (7.5%), pyrexia (6.3%), and capillary leak syndrome (5.0%). Three deaths occurred on study due to pneumonia, septic shock, and sepsis syndrome and underlying HCL; none were considered treatment related. The most common treatment-related adverse events leading to permanent discontinuation were hemolytic uremic syndrome (*n* = 4), capillary leak syndrome (*n* = 2), and blood creatinine increased (*n* = 2; associated with hemolytic uremic syndrome). All hemolytic uremic syndrome and capillary leak syndrome events were reversible. Details regarding hemolytic uremic syndrome/capillary leak syndrome cases are presented in the Supplementary Appendix. Key laboratory findings are presented in Table S2. The median key immunoglobulin (IgA, IgG, and IgM) levels remained unchanged after treatment. Median CD4 T cell counts were stable or increased following a transient decrease on day eight.

**Table 3:** Summary of adverse events^a^

Adverse event	All grades	Grades 3/4
	Patients, *n* (%)	
Edema peripheral	31 (38.8%)	0
Nausea	28 (35.0%)	2 (2.5%)
Fatigue	27 (33.8%)	0
Headache	26 (32.5%)	0
Pyrexia	25 (31.3%)	1 (1.3%)
Hypocalcaemia	19 (23.8%)	0
Hypophosphatemia	19 (23.8%)	8 (10.0%)
Constipation	18 (22.5%)	0
Anemia	17 (21.3%)	8 (10.0%)
Diarrhea	17 (21.3%)	0
Alanine aminotransferase increased	17 (21.3%)	1 (1.3%)
Lymphocyte count decreased	16 (20.0%)	16 (20.0%)
Hypoalbuminemia	16 (20.0%)	0
Hypokalemia	13 (16.3%)	2 (2.5%)
Hypertension	12 (15.0%)	6 (7.5%)
Platelet count decreased	9 (11.3%)	5 (6.3%)
Hyponatremia	9 (11.3%)	2 (2.5%)
White blood cell count decreased	8 (10.0%)	7 (8.8%)
Capillary leak syndrome	7 (8.8%)	2 (2.5%)
Upper respiratory infection	7 (8.8%)	2 (2.5%)
Hemolytic uremic syndrome	6 (7.5%)	4 (5.0%)
Neutrophil count decreased	6 (7.5%)	5 (6.3%)
Febrile neutropenia	5 (6.3%)	4 (5.0%)
Neutropenia	4 (5.0%)	4 (5.0%)
Hypoxia	4 (5.0%)	2 (2.5%)
Lung infection	3 (3.8%)	2 (2.5%)
Acute kidney injury	3 (3.8%)	2 (2.5%)
Erysipelas	2 (2.5%)	2 (2.5%)

^a^Adverse events of any grade with an incidence of at least 20%, as well as events of grade 3 or grade 4 with an incidence of at least 2.5%

### Pharmacokinetics, immunogenicity, and pharmacodynamics

Moxetumomab pasudotox pharmacokinetics were characterized by rapid plasma concentration decline following intravenous administration (Table S3 and Figure S3A in Supplementary Appendix). Exposure was higher after subsequent doses versus the first dose, likely related to treatment-mediated reduction of the CD22 sink (Figure S3B in Supplementary Appendix). Anti-drug antibodies were detected at baseline in 45/76 evaluable patients (59.2%). The frequency of neutralizing antibodies and anti-drug antibody titer increased with repeated cycles of treatment; reduced drug exposure was observed in patients with high-titer (>10 000) anti-drug antibodies (Figures S3C and S3D in Supplementary Appendix). Median peripheral blood CD19 B cell counts (including normal B cells and hairy cells) were reduced by 90% on day eight, remained low through the end of treatment, and, for patients with partial or complete response, returned to approximately normal levels at six months posttreatment (Figure S3E in Supplementary Appendix).

## Discussion

To date, this pivotal study is the largest prospective study in third-line or beyond relapsed/refractory HCL; it is also the first study using durable complete response (defined as complete response assessed by blinded independent central review with maintenance of hematologic remission for more than 180 days) as the primary end point. Patients were heavily pretreated; 50% received three or more prior courses of purine nucleoside analogs and 75% received prior rituximab. The blinded independent central review-assessed complete response rate of 41% represents a substantial improvement over historical controls, and the durable complete response rate of 30% met the primary end point. In HCL, immunohistochemistry staining of a high-quality bone marrow biopsy may provide more consistent minimal residual disease evaluation than flow cytometry of bone marrow aspirates, which depend on a consistent cellular yield [8]. Published data suggest that the extent of minimal residual disease remaining after therapy in HCL is important in predicting long-term outcome [23, 24]. Retrospective analysis of the phase 1 study of moxetumomab pasudotox in a similar patient population of relapsed/refractory HCL showed that patients who were minimal residual disease-negative by immunohistochemistry had extended duration of response (82.7 versus 54.7 months). This result supported the possible predictive value of immunochemistry-based minimal residual disease for long-term outcome. In this study, a majority of patients (27/33) who achieved complete response also achieved negative minimal residual disease by immunohistochemistry. While long-term follow-up is ongoing, the fact that the median duration of complete response was reached for minimal residual disease-positive patients (5.9 months; with limitation of small patient numbers) but has not for minimal residual disease-negative patients indicates that long-term outcome may be improved by clearing minimal residual disease.

Consistent with prior studies of moxetumomab pasudotox and its predecessor molecule BL22 (CAT-3888) [25, 26], in this pivotal study, response rates were higher in patients who did not have splenomegaly or splenectomy (which was associated with high disease burden in the bone marrow; 4 of the 5 patients had 100% involvement at baseline). Three patients with HCL variant were enrolled; all had high disease burden (all had splenomegaly >18 cm and two had lymphocytosis >20/nL) and did not achieve complete response. According to these observations, it may be advisable to initiate moxetumomab pasudotox treatment earlier in relapse when possible in patients with very high disease burden; further studies would be required to determine whether a more intensive treatment regimen might improve response rates in these patients.

Substantial clinical activity was observed despite a high rate of immunogenicity; ~75% of patients had detectable neutralizing antibodies at the end of treatment, regardless of response status. Patients who achieved complete or partial response typically maintained antibody titers below 10,000, and therefore maintained drug exposure for more treatment cycles than patients with stable or progressive disease, suggesting that approaches to reduce the immunogenicity of moxetumomab pasudotox might further improve response.

Hemolytic uremia syndrome and capillary leak syndrome were known toxicities and observed in the moxetumomab pasudotox phase 1 study and in studies of BL22 [25, 27]. In the current study, these events (observed in 10 patients [12.5%]) were typically managed with close monitoring of vital signs and laboratory values (including blood pressure, body weight, blood creatinine, and schistocytes in peripheral blood smear) and supportive medical care (including adequate hydration), with intensive care without plasma exchange and treatment discontinuation for severe cases. Although the exact mechanisms are not well understood, our experience suggests that incidence and severity of hemolytic uremia syndrome and capillary leak syndrome may be reduced by ensuring adequate oral hydration during the first week of each cycle and proper (not excessive) intravenous fluid supplementation on the day of infusion. Dexamethasone may be considered in patients experiencing nausea or fever, to maintain oral hydration for adequate renal perfusion. Worsening renal function was observed in isolation and in association with hemolytic uremia syndrome; based on these findings, close monitoring of renal function is recommended during treatment.The pivotal study data demonstrate that moxetumomab pasudotox provides a deep and durable response with ability to eradicate minimal residual disease in a substantial fraction of patients with relapsed/refractory HCL who have exhausted available therapies, and has a favorable safety profile compared to other available agents. A high percentage of patients were able to receive the full treatment course, and the most important risks, hemolytic uremia syndrome, and capillary leak syndrome, although relatively infrequent, were manageable and reversible with close monitoring and best supportive care. Moxetumomab pasudotox treatment results in substantially less bone marrow suppression than purine nucleoside analogs, without exacerbating baseline infections. In conclusion, moxetumomab pasudotox offers a clinically meaningful treatment for patients with relapsed/refractory HCL.

## Data availability

The data sets generated and/or analyzed during the current study are available from the corresponding author upon reasonable request.

## Electronic supplementary material

Supplementary Appendix
