# Supplementary material for: Moxetumomab pasudotox in relapsed/refractory hairy cell leukemia
Source: Leukemia. 2018 Jul 20;32(8):1768–77. doi: 10.1038/s41375-018-0210-1 (PMC6087717; doi:10.1038/s41375-018-0210-1)
Supplement: Supplementary file 1 — Supplementary Appendix [file 41375_2018_210_MOESM1_ESM.pdf]

Supplementary Appendix

Summary

This supplementary appendix includes additional detail regarding minimal residual disease assessments, patient disposition and per-patient results, results of subgroup analyses of efficacy, and additional safety, pharmacokinetic, pharmacodynamic, and immunogenicity data.

Supplementary Methods

Minimal Residual Disease Assessment

Bone marrow biopsy slides were stained by hemoxylin and eosin and by immunohistochemistry at local study sites and provided to the blinded independent central review vendor. A blinded pathologist evaluated stained slides using an Olympus BX43 microscope with 5 objectives (4×, 10×, 20×, 40×, and oil 100×). The pathologist first reviewed hemoxylin and eosin stained bone marrow biopsy slides; at least 200 cells were counted. In the absence of hairy cells in the hemoxylin and eosin stain, IHC stains were reviewed. CD20 was used primarily to determine presence and percentage of hairy cell involvement; interstitial infiltrative patterns or clusters were used to differentiate hairy cells from normal B cells. Annexin A1 and DBA.44 were used to confirm hairy cells in CD20+ clusters for low-level involvement. CD79a and PAX-5 were supplementary to confirm B cell infiltrates. Samples with no (0%) involvement were qualified as minimal residual disease negative and any involvement was considered minimal residual disease positive.

Supplementary Results

Subgroup Analysis of Efficacy

Results of subgroup analyses of blinded independent central review-assessed durable complete response rate, complete response rate, and objective response rate by baseline demographic characteristics and prior HCL therapies showed no apparent differences among subgroups except that patients at least 65 years of age had a significantly lower durable complete response rate (2/31) than the younger subgroup (22/49). This difference can be at least partly explained by the fact that among the eight elderly patients who had complete responses, four were late-confirmed complete responses. These patients, therefore, have the potential to achieve the criteria for durable complete response post data cutoff date.

Hemolytic Uremia Syndrome/Capillary Leak Syndrome

Ten patients experienced HUS and/or CLS. Seven patients (8.8%) had CLS (grade 2, n=5; grade 4: n=2), 7 patients (8.8%) had HUS (grade 2: n=2; grade 3: n=3; grade 4: n=2) including 1 patient who was adjudicated by the sponsor as having experienced a grade 3 HUS-like event (meeting all criteria for grade 3 HUS except for the requirement of at least 5 schistoscytes/high power field), and 4 patients (5.0%) had both.

Pharmacokinetics

After subsequent doses, on study days 8 and 29 CD19+ B cells were almost fully depleted and a weakened relationship with PK was observed, whereas the relationship was still maintained at the highest B-cell counts. Taken together, these data suggest

that inter-individual variability in PK can be explained at least in part by the differences in baseline CD19+ B cell counts and the magnitude of B-cell depletion posttreatment.

Effect of Immunogenicity on Pharmacokinetics

The potential effect of pre-existing antibodies on PK exposure was evaluated by comparing PK data based on the antidrug antibody status at baseline. With consideration of the observed inter-patient variability in exposure, AUC and C_{max} analyses showed that moxetumomab pasudotox PK during Cycle 1 was similar regardless of antidrug antibody status at baseline. However, the presence of postbaseline antidrug antibodies was associated with statistically significant (*t* test, $p<0.05$) changes in peak exposure in cycle 3 and beyond, consistent with increasing titer levels. The patients who tested ADA-positive with high titers (>10 000) had reduced PK exposure compared to ADA-negative patients (**Figure S3**). Taken together, these data indicate that the observed impact of ADA on PK was due to titer level and not directly related to time.

Table S1. Demographic features, prior therapies, and response to moxetumomab pasudotox

Demographics			Prior Lines — No.			Baseline Disease Characteristics						Disease Response			
Pt.	Age	Sex	Total	PNA	Rituximab	HCL Type	Hgb (g/dL)	ANC (nL)	Plat. (nL)	BM H&E (%)	Spleen (mm)	BICR BOR	INV BOR	Durable CR	CR Ongoing
1	48	M	3	3	0	Classical	12.9	1.0	57	30	140	CR	CR	Y	Y
2	59	M	5	5	0	Classical	12.4	0.5	35	85	152	CR	CR	Y	Y
3	73	M	3	1	2	Classical	12.0	0.1	100	97	129	NE	NE		
4	54	M	3	2	1	Classical	13.1	0.8	64	75	137	CR	CR	Y	Y
5	50	M	2	2	0	Classical	15.3	1.8	89	70	115	CR	CR	Y	Y
6	44	M	2	2	2	Classical	10.1	0.7	101	20	98	PR	CR		
7	47	M	3	2	1	Classical	10.5	0.7	109	75	NE	CR	CR	Y	Y
8	64	M	4	3	1	Classical	10.7	0.8	140	70	109	CR	CR	Y	N
9	67	M	2	2	0	Classical	7.4	0.1	15	95	107	SD	NE		
10	43	F	2	1	1	Classical	11.8	0.5	75	90	110	PR	CR		
11	34	F	4	3	1	Classical	9.0	0.2	26	80	126	PR	PR		
12	52	M	4	3	2	Classical	13.4	0.6	68	20	118	CR	CR	Y	Y
13	56	M	3	2	2	Classical	8.9	0.5	80	NA	121	PR	PR		
14	75	M	3	2	1	Classical	10.7	2.4	60	97	161	PR	PR		
15	71	M	4	3	1	Classical	10.4	0.3	58	85	92	SD	SD		
16	77	M	4	3	0	Classical	12.0	4.3	15	75	209	SD	PD		
17	52	M	3	3	1	Classical	12.7	0.8	71	40	179	CR	CR	Y	N
18	63	M	4	2	1	Classical	10.5	1.1	119	90	122	CR	CR	Y	Y
19	49	M	3	2	1	Classical	12.2	0.7	55	95	170	CR	CR	Y	Y
20	49	M	2	2	0	Classical	10.0	0.8	86	90	106	PR	PR		
21	60	M	2	2	1	Classical	11.4	1.0	71	99	128	CR	CR	Y	N
22	41	F	2	2	0	Classical	9.9	0.9	76	20	130	CR	CR	Y	Y
23	58	M	7	3	4	Classical	11.7	1.0	91	85	97	CR	CR	Y	Y
24	54	M	4	3	1	Classical	14.1	3.7	73	80	121	CR	CR	Y	Y
25	43	F	2	2	1	Classical	9.7	0.4	88	98	100	PR	PR		
26	41	F	4	2	1	Classical	11.6	0.5	109	96	133	CR	CR	N	Y
27	60	M	6	4	1	Classical	11.9	0.6	105	80	148	CR	CR	Y	Y

Demographics			Prior Lines — No.			Baseline Disease Characteristics						Disease Response			
**Pt.**	**Age**	**Sex**	**Total**	**PNA**	**Rituximab**	**HCL Type**	**Hgb (g/dL)**	**ANC (nL)**	**Plat. (nL)**	**BM H&E (%)**	**Spleen (mm)**	**BICR BOR**	**INV BOR**	**Durable CR**	**CR Ongoing**
28	52	M	3	3	0	Classical	10.9	0.9	107	NE	150	CR	PR	N	Y
29	72	M	6	3	2	Classical	13.0	1.6	48	12	103	PR	PR		
30	62	M	4	2	2	Classical	13.6	0.9	50	80	157	CR	CR	Y	Y
31	53	M	2	1	0	Classical	12.3	0.8	84	15	162	CR	CR	Y	Y
32	71	M	2	2	0	Classical	9.6	1.2	75	100	103	CR	PR	N	N
33	68	F	6	5	2	Classical	7.4	0.4	13	99	176	SD	PD		
34	64	F	6	4	1	Classical	12.1	0.8	100	80	134	PR	CR		
35	36	F	3	2	1	Classical	7.9	0.3	90	98	91	PR	PR		
36	65	M	3	3	0	Classical	10.8	2.5	40	NA	187	CR	CR	N	Y
37	67	M	7	6	2	Classical	14.3	0.2	29	85	139	CR	CR	Y	Y
38	56	M	3	1	1	Classical	13.3	0.9	79	65	177	SD	CR		
39	72	M	4	3	1	Classical	7.2	0.8	92	99	156	PR	PR		
40	60	M	4	2	0	Classical	14.2	1.5	51	90	172	CR	PR	N	N
41	82	M	7	3	2	Classical	7.7	2.2	23	100	splenectomy	SD	CR		
42	57	M	9	4	5	Classical	7.4	0.3	19	100	splenectomy	PR	PR		
43	53	F	4	2	0	Classical	9.0	0.2	57	80	135	CR	CR	Y	Y
44	70	M	3	3	0	Classical	10.9	0.7	52	85	180	NE	NE		
45	46	M	3	1	1	Classical	15.1	1.4	55	60	155	CR	CR	Y	Y
46	72	M	5	3	2	Classical	11.6	0.8	50	90	144	PR	PR		
47	52	M	3	3	0	Classical	12.8	1.3	106	70	121	PR	CR		
48	76	M	3	2	1	Classical	11.3	1.1	63	80	133	CR	CR	Y	Y
49	73	M	5	3	1	Classical	9.7	0.7	46	99	147	PR	PR		
50	80	M	4	4	1	Classical	9.4	0.8	29	NE	89	PR	CR		
51	59	F	4	2	1	Classical	9.0	0.4	50	90	159	PR	PR		
52	73	F	3	2	1	Classical	10.6	0.8	72	85	105	CR	PR		Y
53	84	F	3	1	1	Classical	12.3	1.5	60	90	96	CR	CR	N	Y
54	41	M	4	3	1	Classical	14.7	1.4	75	20	147	PR	CR		
55	44	M	2	2	1	Classical	13.8	0.5	99	96	101	CR	CR	Y	N
56	68	M	3	2	1	Classical	12.1	0.6	55	98	125	PR	CR		

Demographics			Prior Lines — No.			Baseline Disease Characteristics						Disease Response			
Pt.	Age	Sex	Total	PNA	Rituximab	HCL Type	Hgb (g/dL)	ANC (nL)	Plat. (nL)	BM H&E (%)	Spleen (mm)	BICR BOR	INV BOR	Durable CR	CR Ongoing
57	45	M	2	1	1	Classical	16.0	0.7	138	90	136	CR	CR	Y	Y
58	62	F	2	1	1	Variant	11.4	6.2	71	40	207	SD	PR		
59	58	M	2	2	0	Classical	12.7	0.6	67	0	133	SD	CR		
60	77	F	6	6	2	Classical	10.7	2.0	350	8	splenectomy	PR	SD		
61	48	M	2	2	1	Classical	10.8	1.0	96	99	133	PR	PR		
62	51	M	9	5	1	Classical	7.4	1.3	17	100	splenectomy	NE	SD		
63	66	F	4	4	0	Classical	9.3	0.7	6	100	splenectomy	SD	SD		
64	79	F	8	5	1	Classical	12.8	4.2	81	75	141	NE	NE		
65	82	M	5	5	2	Classical	9.6	0.9	41	100	99	CR	CR	N	Y
66	47	M	5	2	0	Classical	9.7	0.9	43	100	115	NE	SD		
67	70	M	9	4	3	Classical	11.5	0.4	54	70	105	CR	CR	N	Y
68	64	M	7	4	2	Classical	9.4	0.2	41	70	128	PD	SD		
69	68	M	7	4	2	Classical	9.6	1.1	72	100	102	PR	PR		
70	50	M	3	2	1	Classical	16.3	1.1	87	NE	125	CR	CR	Y	Y
71	58	F	3	3	0	Classical	13.4	0.9	131	40	104	PR	CR		
72	71	M	8	4	1	Classical	8.0	0.9	36	100	170	PR	CR		
73	73	M	5	3	2	Classical	6.5	0.3	12	100	247	NE	NE		
74	54	M	3	3	1	Classical	8.3	0.8	54	95	124	PD	SD		
75	57	M	2	1	1	Classical	11.1	0.4	80	95	157	SD	PD		
76	64	M	2	1	2	Variant	8.0	3.7	90	85	205	PR	SD		
77	69	M	11	7	2	Classical	8.8	0.3	31	NE	136	SD	PR		
78	71	M	2	2	2	Variant	12.9	2.9	68	0	182	SD	SD		
79	62	M	7	4	1	Classical	11.1	0.4	54	100	132	PR	PR		
80	66	M	2	2	0	Classical	12.6	0.8	70	50	198	PR	PR		

Table S2. Summary of Changes in Laboratory Values

Parameter	Baseline<br>Median (Minimum, Maximum)	Worst on Treatment<br>Median (Minimum, Maximum)	End of Treatment<br>Median (Minimum, Maximum)
Hemoglobin, g/dL	11.10 (6.5, 16.3)	10.10 (6.4, 14.2)	13.14 (7.2, 16.5)
Neutrophils, nL	0.81 (0.1, 6.2)	0.55 (0.0, 3.2)	2.75 (0.1, 7.0)
Platelets, nL	68 (6, 350)	63 (5, 263)	162 (7, 439)
CD4+ T cells, nL	350 (36, 1 500)	222 (21, 683)	390 (57, 2 370)
IgA, mg/dL	106.5 (11, 260)	95.0 (9, 250)	105.0 (6,339)
IgG, mg/dL	834.0 (387, 3 003)	801.0 (237, 2 664)	855.0 (237, 3 075)
IgM — mg/dL	42.0 (6, 327)	35.0 (5, 237)	49.0 (5, 312)
Creatinine, mg/dL	0.85 (0.4, 1.3)	1.11 (0.6, 5.6)	0.96 (0.5, 4.3)

Table S3. Pharmacokinetic Parameters

Parameter (units)	Cycle 1, Day 1	Cycle 1, Day 5	Cycle 2, Day 1
T_{max}, h	0.567 (0.433–1.30) [75]	0.550 (0.417–2.45) [71]	0.583 (0.500–1.75) [69]
C_{max}, ng/mL	192 (162) [75]	435 (233) [71]	379 (262) [69]
$AUC_{0\text{-}last}$, ng.h/mL	120 (261) [75]	820 (721) [71]	626 (610) [69]
$AUC_{0\text{-}inf}$, ng.h/mL	NR*	1300 (742) [49]	1470 (541) [22]
CL, mL/h/kg	NR*	44.6 (30.5) [49]	31.8 (13.7) [22]
$t_{½}$, h	NR*	1.38 (0.632) [49]	1.39 (0.351) [22]

*Not reported owing to lack of measurable concentration in the terminal phase.

Note: Values presented as mean (standard deviation) [N], except for T_{max}, which is shown as median and range (minimum–maximum). All pharmacokinetic parameters are rounded to 3 significant figures.

Figure S1. Patient Disposition

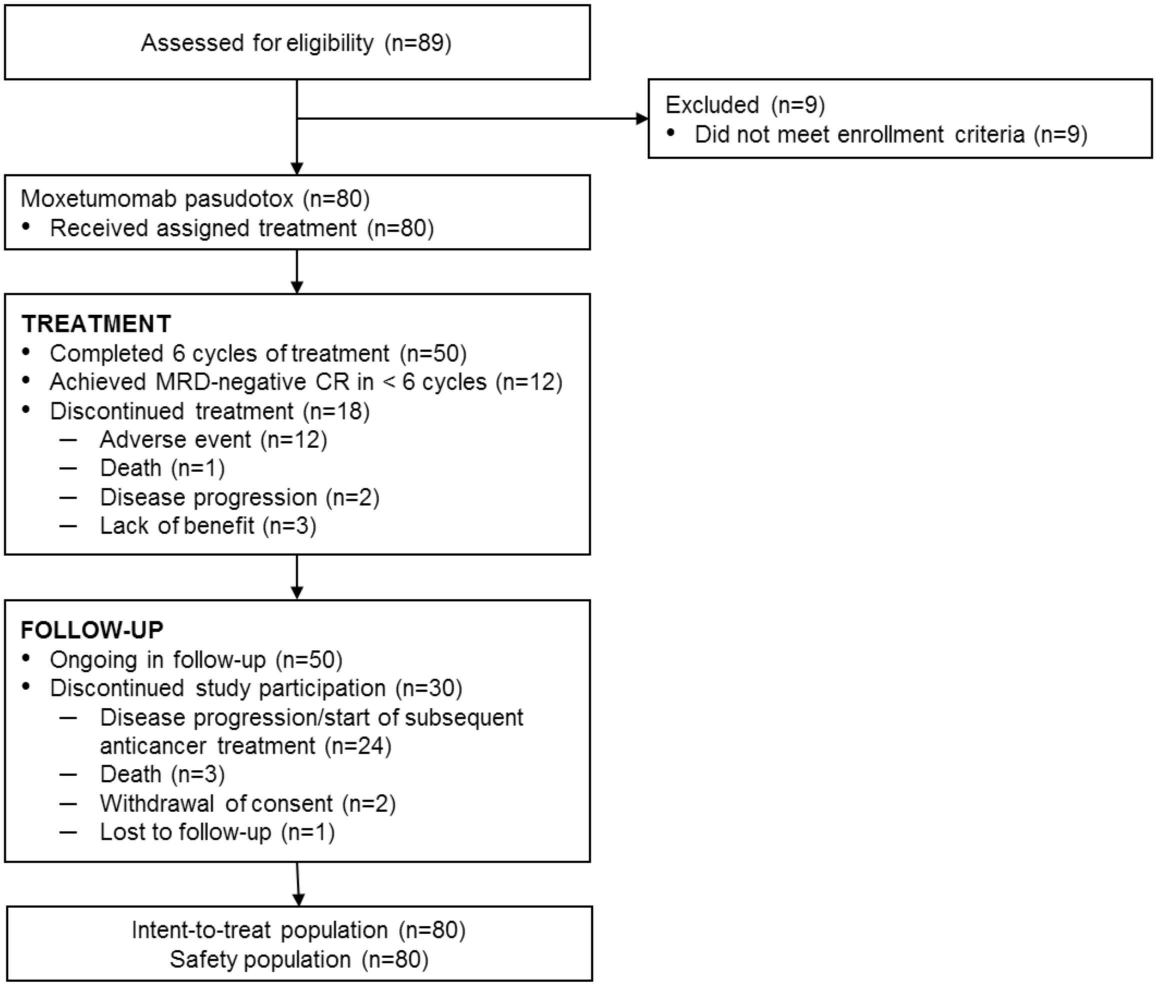

Figure S2. Forest plot of complete response by subgroup.

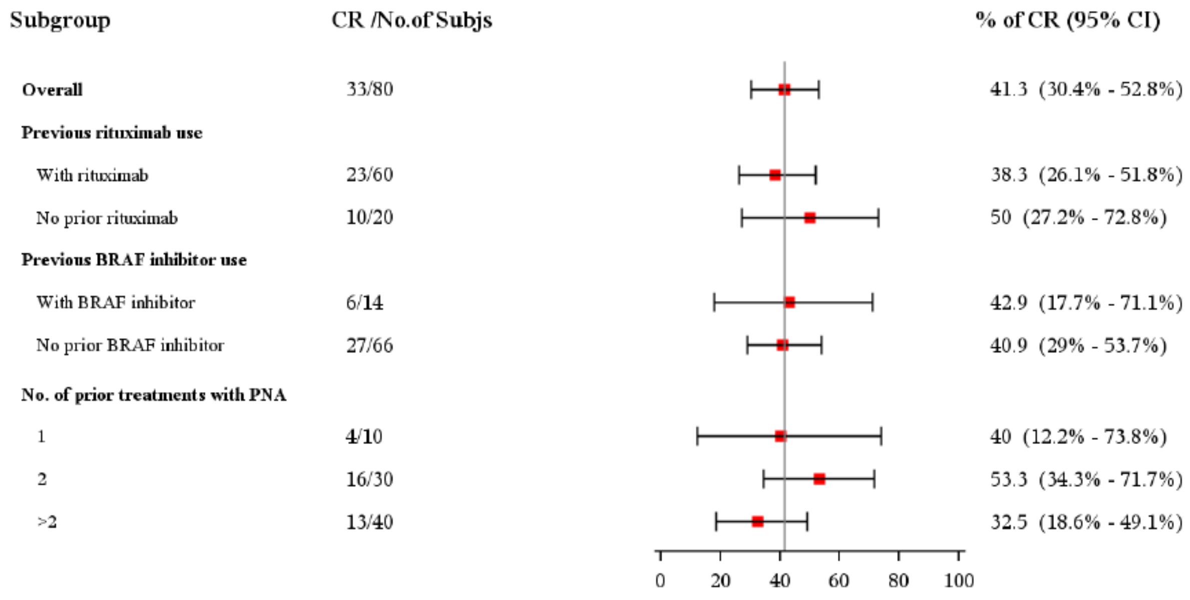

Figure S3. Pharmacokinetics, immunogenicity, and pharmacodynamics. A. Mean concentration-time profiles following administration in the first two cycles of treatment. Error bars represent standard deviation of the mean. Data below the lower limit of quantification (40 ng/mL; as shown by dotted horizontal line) are plotted at 20 ng/mL for illustrative purposes only. B. Correlation between baseline B cell counts and moxetumomab pasudotox exposure following the first dose. Symbols are individual observations; solid line represents line of fit; shaded area represents the 95% confidence interval for the mean line of fit; ln=natural logarithm. C. Percent of patients with neutralizing antibodies and anti-drug antibody titer as a function of time and best objective response. Median and interquartile range are shown. Drug exposure was found to be reduced when anti-drug antibody titer was high (>10 000); this threshold is noted with a dashed line. D. Effect of anti-drug antibody titer on moxetumomab pasudotox exposure. Symbols are individual observations; solid line represents the LOESS smoothed fit; shaded area represents the 95% confidence interval for the line of fit. E. CD19+ B cell count as a function of time and best objective response. Median and interquartile range are shown. Note that one patient (best objective response of partial response, achieved 6 months post end of treatment) with very high B cell counts (>50 000 cells/µL) throughout treatment was excluded from this figure.

A.

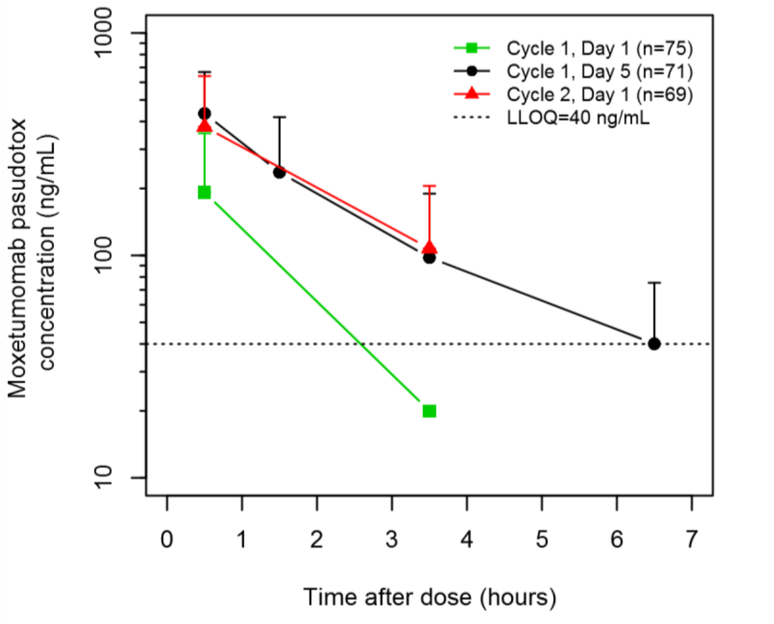

B

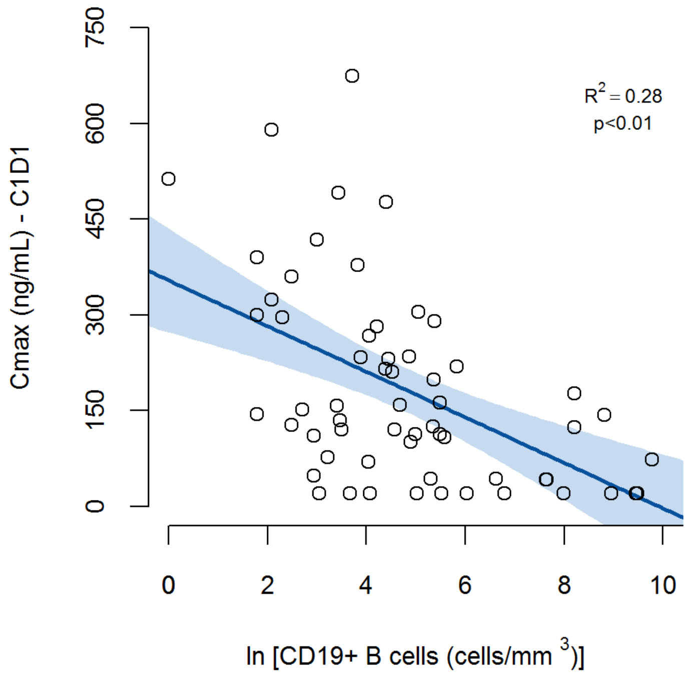

C.

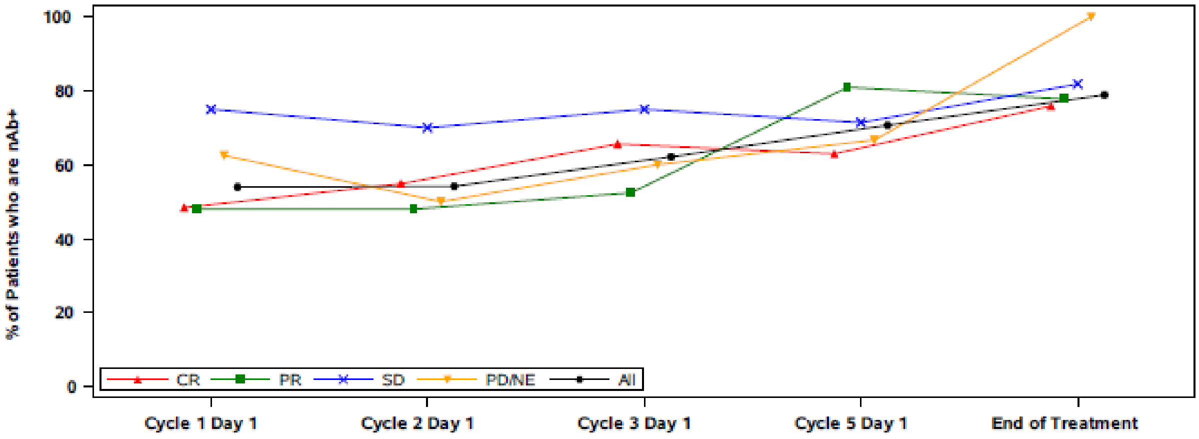

CR	N = 31	N = 31	N = 32	N = 27	N = 29
PR	N = 25	N = 25	N = 21	N = 21	N = 27
SD	N = 12	N = 10	N = 8	N = 7	N = 11
PD/NE	N = 8	N = 6	N = 5	N = 3	N = 4
All	N = 76	N = 72	N = 66	N = 58	N = 71

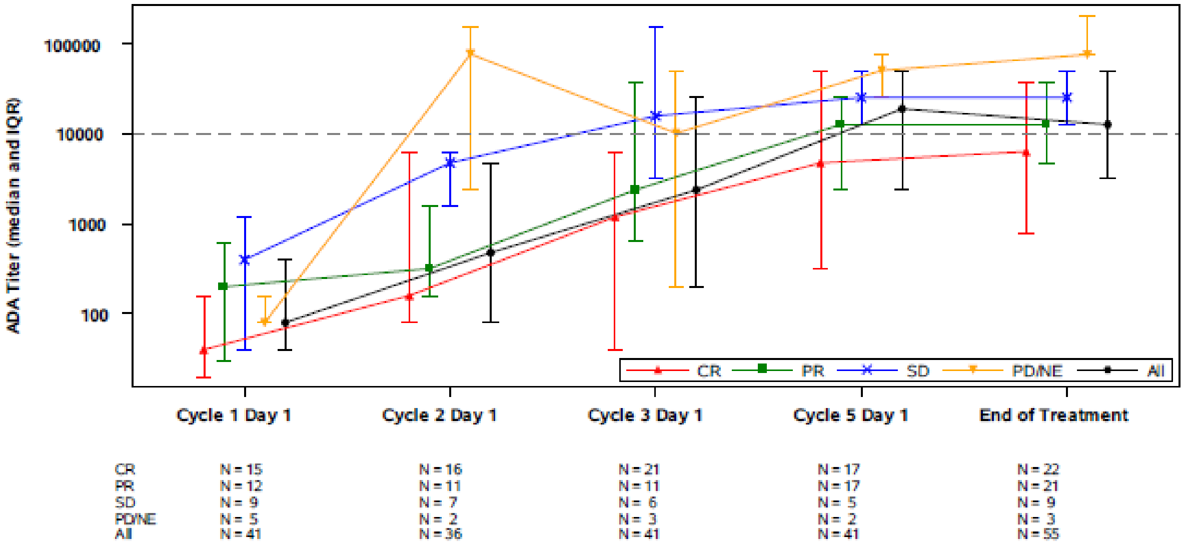
ADA Titer (median and IQR)
100000
10000
1000
100
0
CR
PR
SD
PD/NE
All
Cycle 1 Day 1
Cycle 2 Day 1
Cycle 3 Day 1
Cycle 5 Day 1
End of Treatment
CR N = 15 N = 16 N = 21 N = 17 N = 22
PR N = 12 N = 11 N = 11 N = 17 N = 21
SD N = 9 N = 7 N = 6 N = 5 N = 9
PD/NE N = 5 N = 2 N = 3 N = 2 N = 3
All N = 41 N = 36 N = 41 N = 41 N = 55

D.

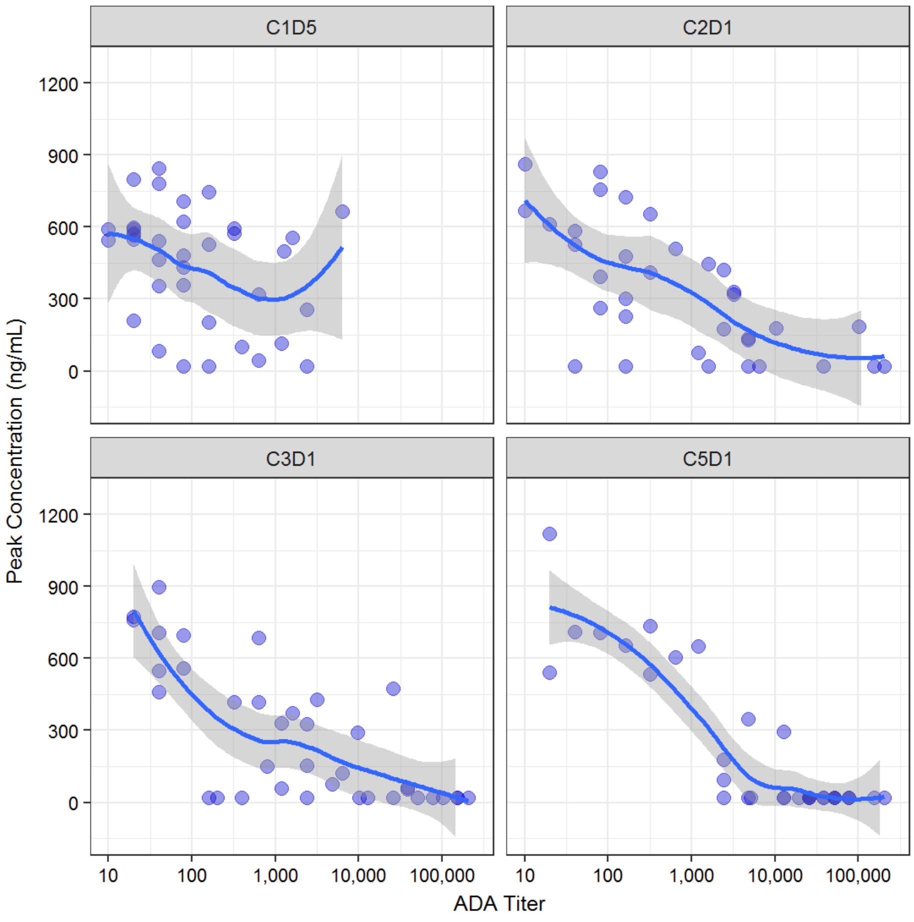
C1D5
C2D1
C3D1
C5D1
Peak Concentration (ng/mL)
1200
900
600
300
0
10
100
1,000
10,000
100,000
ADA Titer

E.

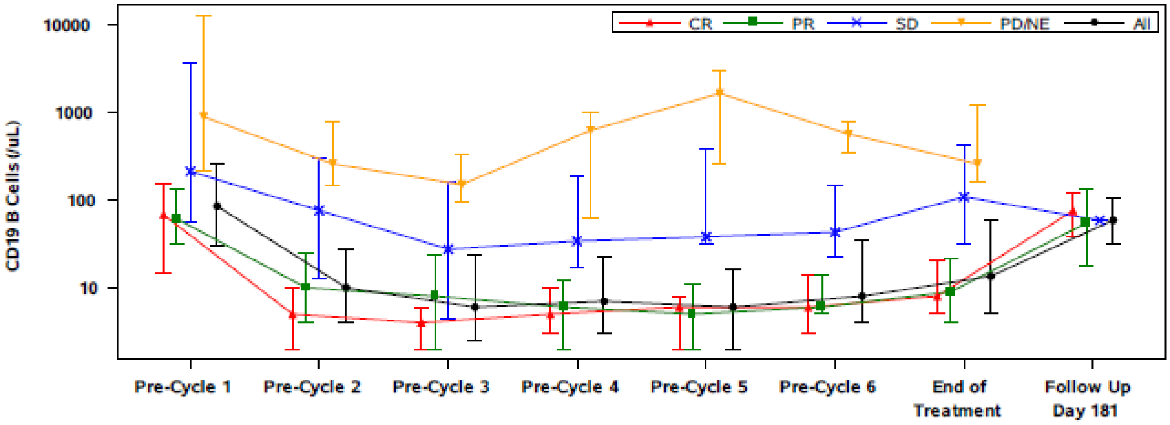

	Pre-Cycle 1	Pre-Cycle 2	Pre-Cycle 3	Pre-Cycle 4	Pre-Cycle 5	Pre-Cycle 6	End of Treatment	Follow Up Day 181
CR	N = 25	N = 23	N = 27	N = 23	N = 25	N = 21	N = 25	N = 20
PR	N = 22	N = 22	N = 20	N = 22	N = 21	N = 21	N = 26	N = 16
SD	N = 9	N = 9	N = 8	N = 8	N = 5	N = 7	N = 10	N = 2
PD/NE	N = 7	N = 4	N = 5	N = 3	N = 2	N = 2	N = 3	
All	N = 63	N = 58	N = 60	N = 56	N = 53	N = 51	N = 64	N = 38
